# Supplementary material for: Mitochondrial Fatty Acid β-Oxidation and Resveratrol Effect in Fibroblasts from Patients with Autism Spectrum Disorder
Source: J Pers Med. 2021 Jun 4;11(6):510. doi: 10.3390/jpm11060510 (PMC8229571; doi:10.3390/jpm11060510)
Supplement: Supplementary file 1 [file jpm-11-00510-s001.zip › jpm-1228025-supplementary.pdf]

Journal of *Personalized Medicine*

MDPI

Mitochondrial fatty acid β-oxidation and resveratrol effect in fibroblasts frompatients with autism spectrum disorder

Supplementary information

Table S1. Standardized clinical assessment scores in participants with autism spectrum disorder (ASD). Values are provided for the entire group (ASD (all)) as well as participants with ASD that also have acyl-carnitine (AC) elevations (ASD w/-AC) and participants with ASD without AC elevations (ASD w/o-AC). The mean difference between groups is provided and bolded and italic if it exceeds the mean clinically important difference (MCID), indicating that the difference would be expected to be noticed clinically.

	**ASD (all)** *n* = **10**	**ASD (w/-AC)** *n* = **5**	**ASD (w/o-AC)** *n* = **5**	**Mean Difference**	**MCID**	***t, p***
Age (years)	7.4 ± 3.2	7.2 ± 3.7	7.6 ± 3.1	0.3		0.35, ns
Vineland Adaptive Behavior Scale						
Daily living Skills	70.1 ± 16.3	73.8 ± 12.2	66.5 ± 18.1	*7.3*	2.8	0.42, ns
Communication Skills	73.1 ± 16.3	68 ± 15.6	78.3 ± 15.3	*10.3*	3.8	0.59, ns
Socialization Skills	70.5 ± 16	66.8 ± 10.3	73.3 ± 18.1	*6.5*	3.0	0.66, ns
Aberrant Behavior Checklist						
Irritability	17 ± 10.7	13.8 ± 13.2	19.6 ± 8.9	*5.8*	1.8	0.45, ns
Social Withdrawal	12.9 ± 7.8	17.5 ± 7.9	9.2 ± 6.1	*8.3*	1.0	0.12, ns
Hyperactivity	20.7 ± 13.1	18.8 ± 15.2	22.2 ± 12.8	*3.4*	2.6	0.72, ns
Stereotypies	5.6 ± 5.5	8.8±5.6	3.1 ± 4.5	*5.7*	0.7	0.12, ns
Inappropriate Speech	3.6 ± 4.4	5.3 ± 5.6	2.2 ± 3.2	*3.1*	0.7	0.33, ns
Social Responsiveness Scale						
Awareness	73.4 ± 11.5	69.6 ± 10.5	77.2 ± 12.3	*7.6*	7.1	0.32, ns
Cognition	71.6 ± 13.5	68.6 ± 12.2	74.6 ± 15.4	*6.0*	5.8	0.51, ns
Communication	76.2 ± 14.3	77 ± 14.7	75.4 ± 15.6	1.6	4.2	0.87, ns
Motivation	73.5 ± 14.7	72.6 ± 14.3	74.4 ± 16.8	1.8	5.7	0.86, ns
Mannerism (RRB)	79.2 ± 11.4	83.6 ± 10.4	74.8 ± 11.7	*8.8*	5.5	0.24, ns
Total	78.4 ± 13.5	79 ± 13.5	77.8 ± 15	1.2		0.90, ns

RRB: restricted interests and repetitive behavior.

Table S2. Correlation analysis (*r*) among clinical severity and mitochondrial fatty acid oxidation (mtFAO) activity in fibroblasts from patients with ASD under basal conditions and after resveratrol (RSV+) treatment.

	**mtFAO**		
Clinical characteristics	Baseline	RSV +	Δ
VABS Communication	0.59	-0.08	−0.36
VABS Daily Living Skills	0.15	-0.47	−0.65
VABS Socialization	0.41	-0.38	−0.65
ABC Irritability	0.28	0.38	0.28
ABC Social Withdrawal	−0.62	0.08	0.42
ABC Hyperactivity	−0.13	0.12	0.21
ABC Stereotypic Behavior	−0.66	−0.03	0.31
ABC Inappropriate Speech	−0.24	0.24	0.39
SRS Awareness	−0.16	0.72 *	0.89 ***
SRS Cognition	−0.05	0.76 *	0.88 ***
SRS Community	−0.34	0.54	0.78 **
SRS Motivation	−0.15	0.61	0.77 **
SRS Mannerism (RRB)	−0.59	0.28	0.63
SRS Total	−0.27	0.65 *	0.87 ***

VABS: Vineland Adaptive Behavior Scale; ABC: Aberrant Behavior Checklist; SRS: SocialResponsiveness Scale; RRB: restricted interests and repetitive behavior. *: $p \leq 0.05$; **: $p \leq 0.01$; ***: $p \leq 0.00$
